# Biomimetic Cationic Nanoparticles Based on Silica: Optimizing Bilayer Deposition from Lipid Films

**DOI:** 10.3390/biomimetics2040020

**Published:** 2017-10-20

**Authors:** Rodrigo T. Ribeiro, Victor H. A. Braga, Ana M. Carmona-Ribeiro

**Affiliations:** Biocolloids Laboratory, Instituto de Química, Universidade de São Paulo, Av. Lineu Prestes 748, São Paulo 05508-000, SP, Brazil; rodrigo@iq.usp.br (R.T.R.); victor.hugo.braga@usp.br (V.H.A.B.)

**Keywords:** AEROSIL OX-50, dioctadecyldimethylammonium bromide, *N*-[1-(2,3-dioleoyloxy)propyl]-*N*,*N*,*N*-trimethylammonium chloride, elemental analysis for in situ adsorption, colloidal stability, cationic bilayer fragments, films of cationic lipids, optimal bilayer adsorption from films

## Abstract

The optimization of bilayer coverage on particles is important for a variety of biomedical applications, such as drug, vaccine, and genetic material delivery. This work aims at optimizing the deposition of cationic bilayers on silica over a range of experimental conditions for the intervening medium and two different assemblies for the cationic lipid, namely, lipid films or pre-formed lipid bilayer fragments. The lipid adsorption on silica in situ over a range of added lipid concentrations was determined from elemental analysis of carbon, hydrogen, and nitrogen and related to the colloidal stability, sizing, zeta potential, and polydispersity of the silica/lipid nanoparticles. Superior bilayer deposition took place from lipid films, whereas adsorption from pre-formed bilayer fragments yielded limiting adsorption below the levels expected for bilayer adsorption.

## 1. Introduction

Biomimetic coatings on a variety of inorganic [1,2,3] or polymeric nanoparticles (NPs) [4,5,6] bear the potential for optimal delivery of drugs [7,8], nucleic acids [9,10], or vaccines to biological cells [11,12,13]. The interaction between nanoparticles (NPs) and lipids in water dispersions yielded the so-called biomimetic NPs: bilayer-covered nanostructures for biomedical applications [14,15]. These nanostructures efficiently isolated and reconstituted recognition events that take place at the surface of biological cells, such as specific receptor–ligand interactions and binding of pathogenic toxins to their transmembrane receptor proteins [16,17,18,19,20]. Coverage of colloidal particles with lipids allowed NPs functionalization with a variety of different biomolecules, such as glycolipids, peptides, receptors, proteins, nucleotides, and nucleic acids [18,19]. For example, coverage of silica NPs with a phosphatidylcholine (PC) bilayer allowed for the incorporation of the cholera toxin receptor with isolation and successful reconstitution of the receptor–ligand interaction between monosialoganglioside GM1 and its cholera toxin ligand [15,19]. In order to promote the interaction between lipids and particles with water as the intervening medium, a preliminary step has been dispersing the lipid in water solution as bilayers. Among the lipids, the cationic ones are particularly useful since they easily combine with negatively charged peptides, proteins, nucleic acids, and drugs [21].

In this work, aiming at the optimization of cationic lipid bilayer coating on silica, the adsorbed amount onto silica of two different cationic lipids, dioctadecyldimethylammonium bromide (DODAB) and *N*-[1-(2,3-dioleoyloxy)propyl]-*N*,*N*,*N*-trimethylammonium chloride (DOTAP), from lipid films was determined in situ by means of an elemental analysis of the silica particles, and compared with the one obtained from pre-formed cationic bilayers in dispersion, such as the bilayer fragments (BF). Whereas, DODAB BF were extensively characterized and used before in drug and vaccine delivery [14,15,19,22,23,24,25], DOTAP BF were neither obtained previously nor properly characterized as such. Therefore, in the present work, the interaction between DODAB films and silica particles was compared to the already reported interaction between silica and DODAB BF.

The results showed that the interaction between silica and cationic lipid films led to optimal bilayer adsorption in comparison to the reduced adsorption from previously dispersed DODAB bilayers. This was understood as being due to the occurrence of hydrophobic defects in the pre-formed bilayers that diminished their interaction with the hydrophilic silica NPs.

## 2. Materials and Methods

### 2.1. Materials

**Figure 1:**
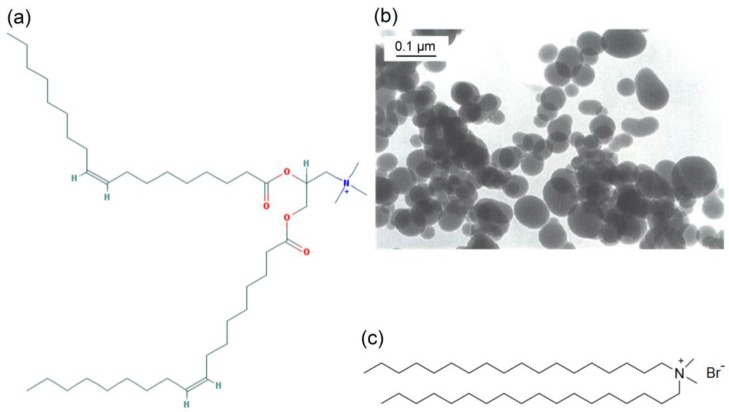
Chemical structures for the cationic lipids and silica micrograph. (**a**) *N*-[1-(2,3-dioleoyloxypropyl)-*N*,*N*,*N*-trimethylammonium chloride (DOTAP) chemical structure. (**b**) Silica particles (AEROSIL OX-50) from transmission electron microscopy (TEM) as provided by the supplier. (**c**) Dioctadecyldimethylammonium bromide (DODAB) chemical structure.

DODAB, DOTAP and KCl were purchased from Sigma (St. Louis, MO, USA). Silica (AEROSIL OX-50) was purchased from Degussa (Frankfurt, Germany). The mean particle diameter determined by the supplier using transmission electron microscopy (TEM) was 50 nm. Specific surface area was previously determined from Brunauer–Emmett–Teller (BET) method yielding 26.00 m^2^/g [25]. The silica dispersion was prepared by dispersing AEROSIL OX-50 in ultrapure water or in a 1mM KCl aqueous solution [26]. Lipids chemical structure and silica micrograph from the suppliers, Sigma and Degussa, respectively, are on Figure 1.

### 2.2. Preparation of Lipid Films

**Figure 2:**
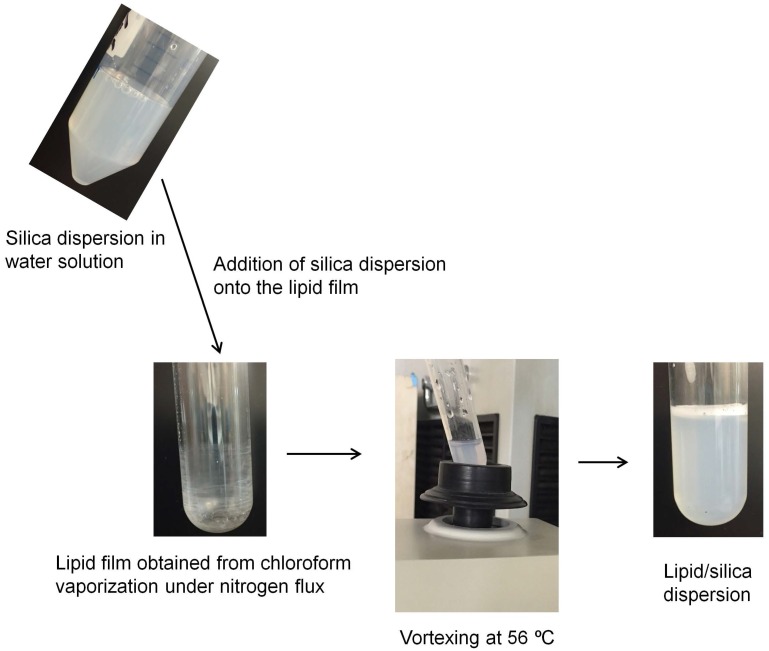
Procedure for dispersing silica/cationic lipid from lipid films. The lipid employed was either DOTAP or DODAB. Vortexing was done at an arbitrary temperature of 56 °C, above the mean phase transition temperature of the DODAB or DOTAP bilayer.

The DODAB or DOTAP chloroform solutions yielded the lipid films on the bottom of glass tubes from chloroform vaporization under a nitrogen flux (Figure 2). Any temperature above the gel-to-liquid crystalline phase transition temperature of DODAB bilayer would yield the interaction between the silica surface and the DODAB bilayer in the liquid crystalline state. Nascimento et al. first obtained the mean temperature of the main phase transition at 47–49 °C [27]; therefore, the choice of 56 °C was arbitrary and defined by our water bath that kept the temperature constant at 56 °C.

### 2.3. Preparation of DODAB Bilayer Fragments

The DODAB BF were obtained from the DODAB powder added to water or 1 mM KCl water solution dispersed by sonication with tip at 85 W for 15 min above 47 °C, before centrifuging the dispersion for precipitation of titanium ejected from the tip (9300× *g* for 1 h at 4 °C). This yielded the DODAB BF [28]. Sonication with a macrotip was performed at room temperature but the procedure heated up the samples under sonication above the mean phase transition temperature. The ultrasonic dispersion of the DODAB powder increased continuously the sample temperature and it was not possible to define a single temperature value while performing the sonication.

### 2.4. Preparation and Characterization of the Biomimetic, Cationic Silica Particles

**Figure 3:**
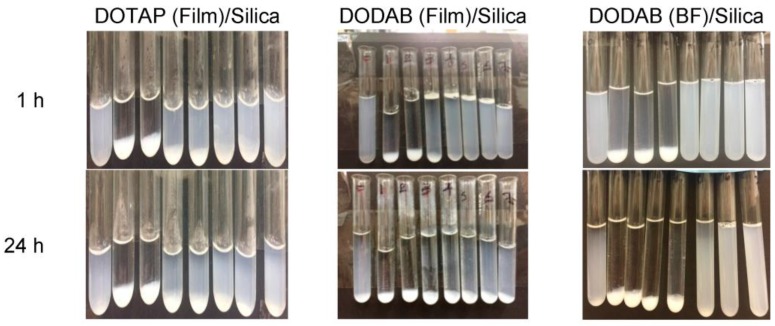
Colloidal stability of silica/cationic lipid dispersions in 1 mM KCl solution over a range of DODAB or DOTAP concentrations at 2 mg/mL silica. DOTAP or DODAB concentrations were 0, 0.05, 0.1, 0.2, 0.3, 0.5, 0.6, and 1.0 mM. BF: Bilayer fragments.

Films or dispersions of the cationic lipids interacted with the silica particles (2 mg/mL) for 1 h at 56 °C before determination of colloidal stability at 1 and 24 h from photos (see Figure 3).

Size, size distribution and zeta potential were measured after 24 h interaction between silica and films or BF, and only for the supernatants diluted in 1 mM KCl or in pure water. Precipitates did not allow the characterization measurements. The characterization of the silica/lipid particles was performed by means of dynamic light scattering (DLS) using a Brookhaven ZetaPlus-ZetaPotential Analyzer (Brookhaven Instruments Corp., Holtsville, NY, USA), equipped with a 677 nm laser and a correlator for DLS at 90° plus software for z-average diameter (*Dz*), zeta potential (*ζ*) and polydispersity (*P*) determinations [26,29]. The zeta potential was obtained from the electrophoretic mobility *μ* and the Smoluchowski equation: *ζ = μη*/*ε*, where *η* is the medium viscosity and *ε* is the dielectric constant of the medium. Measurements for silica or silica/lipid were obtained after diluting the dispersions by 1:20. The algorithm used by the Brookhaven apparatus to calculate the size distributions always tries to solve the size distributions with several peaks [29]. On the other hand, the log normal or Gaussian size distribution fits the intensity of the light scattered to produce only one peak with a mean value. Therefore, the comparison between mean sizes represented a better way of evidencing the reproducibility of physical properties for the dispersions. Precipitation due to interparticle aggregation hampered the determination of physical properties of the NPs by DLS. Again, only the dispersed NPs were used to determine physical properties by this technique. The hydrodynamic radius was determined by the software of the Brookhaven apparatus by a mathematically well-defined algorithm contained in the Grabowski and Morrison chapter on quasi-elastic light scattering [29]. The number of repeats performed by the apparatus was at least 10 determinations. Usually this was done from 20 to 30 determinations for obtaining the mean *Dz*.

### 2.5. Determination of Lipid Adsorption onto Silica from Elemental Analysis of C, H and N

Silica (2 mg/mL) and lipid (0–2 mM) interacted for 24 h before centrifuging (1200× *g* for 1 h), withdrawing the supernatant and lyophilizing the precipitates for performing the elemental analysis using an Elemental Analyzer Perkin Elmer 2400 Series II (Waltham, MA, USA). Thereby, carbon (C), hydrogen (H), and nitrogen (N) elements at the silica surface were analyzed to yield C, H, and N percentages in the samples [30]. These were converted to molar concentration of adsorbed lipid and plotted as a function of the added lipid concentration in each sample. The adsorption of nitrogen (determined through BET) onto silica yielded 26 m^2^/g [25]. The lipids apparently had access to the pores of the silica nanoparticles similarly to the accessibility of the pores to the nitrogen gas [25], as shown from the limiting adsorption of cationic lipids onto silica (see the Results section ahead). At least two aliquots from the interacting mixtures were taken for elemental analysis of the three elements in duplicate for all adsorption isotherms obtained.

## 3. Results

### 3.1. Colloidal Stability of Silica Nanoparticles in the Presence of Cationic Lipids

The interaction between silica (2 mg/mL) and cationic lipid over a range of concentrations (0–2 mM) yielded extensive precipitation over a low range of lipid concentrations (0–0.3 mM); above 0.3 mM lipid, the colloidal stability improved and particles remained in dispersion though some precipitation still occurred over the long run (Figure 3). The colloidal stability for the system can be understood as a lack of aggregation and sedimentation.

This behavior was previously obtained for the interaction between DODAB BF and silica under similar experimental conditions [26]. It is important to notice that the lipid concentrations required for coating all of the silica particles at 2 mg/mL silica with one classical bilayer are 0.288 mM lipid for DODAB and 0.266 mM lipid for DOTAP. This was calculated from the specific surface area for silica, determined through BET as 26 m^2^/g, the silica concentration (2 mg/mL), and the mean molecular area for the lipid at the air–water interface taken as 0.6 nm^2^ for DODAB [31] and 0.65 nm^2^ for DOTAP [32]. The comparison between colloidal stabilities for silica/DODAB from films and silica/DODAB BF revealed a similar behavior (Figure 3).

### 3.2. Physical Properties of Silica/DODAB and Silica/DOTAP Nanoparticles

**Figure 4:**
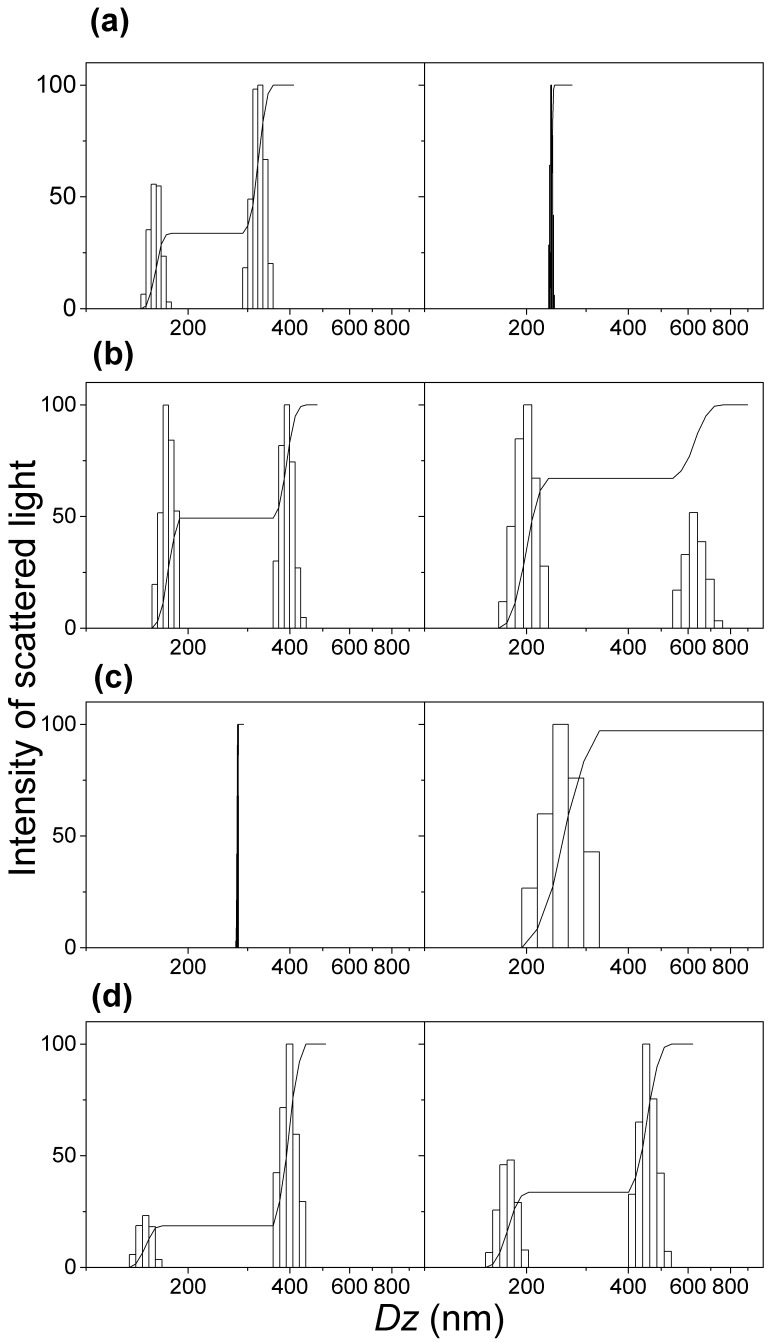
Size distributions for two different but equivalent dispersions: (**a**) silica (2 mg/mL); (**b**) silica/DODAB from DODAB BF at 0.5 mM DODAB; (**c**) silica/DOTAP dispersions from DOTAP films at 0.5 mM DOTAP; (**d**) silica/DODAB from DODAB films at 0.5 mM DODAB. Silica and lipid interacted for 24 h in 1 mM KCl solution before taking the supernatants and diluting them by 1:20 for sizing. *Dz*: Z-average diameter.

The stable silica/lipid NPs available at 0.5 mM lipid had their physical properties, such as histograms for size distributions, *Dz*, *ζ*, and *P* determined from dynamic light scattering (Figure 4 and Table 1).

The reproducibility of the dispersions for equivalent but different samples can be evaluated from Table 1. Silica/lipid samples were very reproducible yielding similar mean *Dz*, *ζ*, and *P*. DOTAP yielded superior silica/DOTAP dispersions when considering the narrow size distributions obtained for silica/DOTAP from DOTAP films (Figure 4). The coverage of silica with DODAB or DOTAP produced, as expected, cationic NPs with high and positive *ζ* (45–56 mV) (Table 1). In particular, the silica/DOTAP dispersions exhibited *P* that were lower than the silica/DODAB ones (Table 1), consistently with the narrow size distributions for silica/DOTAP (Figure 4).

The anionic silica dispersions became cationic upon coverage with the cationic lipids DODAB or DOTAP (Table 1). The zeta potentials for silica/DODAB from DODAB films were higher than the zeta potentials for silica/DODAB from DODAB BF (Table 1). Evidences for interdigitation for cationic bilayers in pure water or at a very low ionic strength were previously reported in the literature from fraction of inner aqueous compartment of cationic vesicles in pure water and molecular dynamics simulations for the bilayer [21,33,34]. The interdigitated DODAB bilayers with hydrophobic defects would not interact so well with silica and once adsorbed onto silica would result in lower zeta potentials than for those obtained for classical bilayers. Interdigitation might be an explanation for the lower zeta potentials for silica/DODAB NPs obtained from pre-formed DODAB BF (Table 1). The interaction between silica and lipid films yielded NPs with higher zeta potentials than those resulting from the interaction between silica and the bilayer fragments (Table 1), showing a higher affinity between DODAB molecules from films than DODAB molecules from BF. Zeta potentials for silica/DOTAP from DOTAP films were similar to those for silica/DODAB from DODAB films (Table 1).

### 3.3. Adsorption Isotherms for DOTAP or DODAB onto Silica from Elemental Analysis In Situ

**Figure 5:**
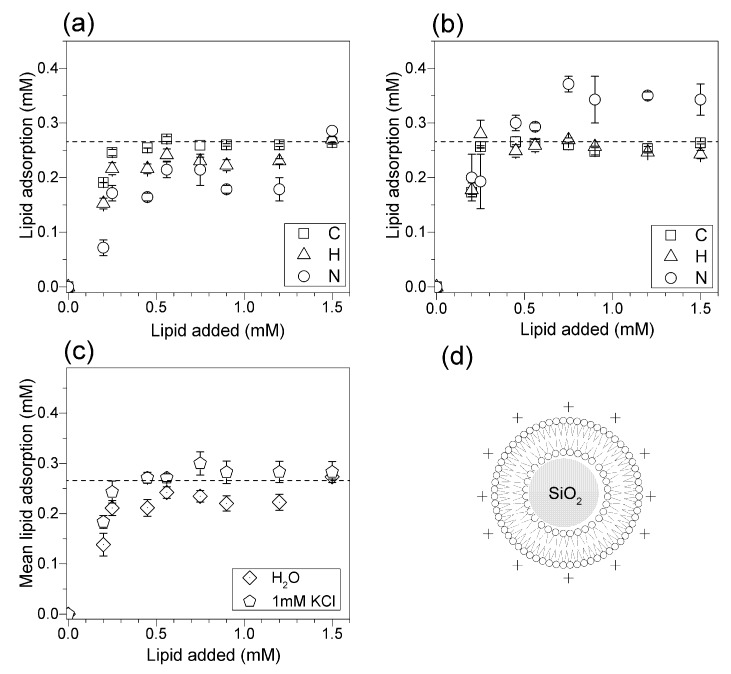
Adsorption isotherms for DOTAP onto silica. (**a**) Adsorption isotherms for DOTAP onto silica from DOTAP films in pure water. (**b**) Adsorption isotherms for DOTAP onto silica from DOTAP films in 1 mM KCl. (**c**) Mean DOTAP adsorption onto silica from DOTAP films in water or in KCl 1 mM aqueous solution. (**d**) Cross-section of a silica NP covered by a DOTAP cationic bilayer. The dashed line at 0.266 mM DOTAP represents the theoretical concentration for bilayer adsorption. Silica concentration was 2 mg/mL. Each mean adsorption value and the respective mean standard deviation was obtained from at least two different determinations for each element.

The elemental analysis of C, H, and N adsorbed on silica allowed a precise and reliable evaluation of the lipid concentration adsorbed onto the silica NPs as a function of the added lipid concentration. DOTAP adsorption on silica was determined in pure water (Figure 5a) or in 1 mM KCl (Figure 5b). The dashed line shows the concentration corresponding to the theoretical DOTAP bilayer adsorption (0.266 mM DOTAP). The isotherm revealed mean limiting adsorption practically equal to this theoretical value as achieved from silica and DOTAP films in 1 mM KCl (Figure 5c). In pure water, the mean limiting DOTAP adsorption was slightly below the theoretical bilayer adsorption (Figure 5c). Possibly, DOTAP adsorbed bilayer also had some interdigitated regions in pure water. Elemental analysis was more reliable for the most abundant elements, since carbon and hydrogen from the lipids could be determined at the silica surface with smaller error bars than the nitrogen determinations (Figure 5).

**Figure 6:**
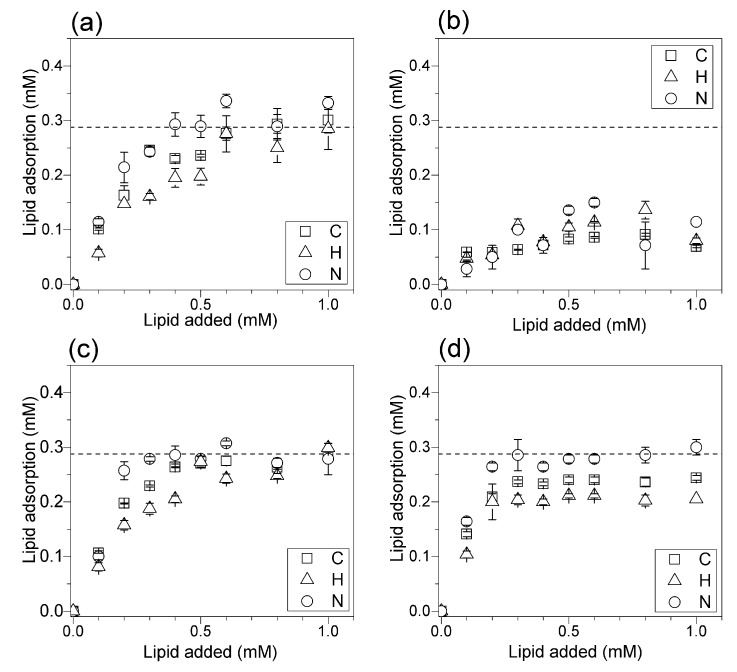
Adsorption isotherms for DODAB onto silica. (**a**) DODAB adsorption onto silica (2 mg/mL) from DODAB films in pure water. (**b**) DODAB adsorption on to silica (2 mg/mL) from DODAB BF in pure water. (**c**) DODAB adsorption onto silica (2 mg/mL) from films in 1 mM KCl water solution. (**d**) DODAB adsorption onto silica (2 mg/mL) from DODAB BF in 1 mM KCl aqueous solution. The dashed line at 0.288 mM DODAB represents the theoretical concentration for bilayer adsorption. Each mean adsorption value and respective mean standard deviation was obtained from at least two different determinations for each element.

DODAB adsorption onto silica from films (Figure 6a,c) or from DODAB BF (Figure 6b,d) was determined in pure water (Figure 6a,b) or in 1 mM KCl aqueous solution (Figure 6c,d). The mean limiting adsorption for DODAB from films yielded bilayer deposition both in pure water (Figure 6a) and in 1 mM KCl over a range of DODAB concentrations above 0.3 mM DODAB (Figure 6c). DODAB adsorption from films yielded bilayer deposition (Figure 6a,c) in contrast to the adsorption from BF, which did not reach adsorbed amounts corresponding to bilayer deposition (Figure 6b,d). In pure water or in 1 mM KCl, DODAB adsorption from BF was lower than the one expected for deposition of a classical bilayer (Figure 6b,d). Thus, adsorption from films optimized the coating on silica with a classical bilayer.

**Figure 7:**
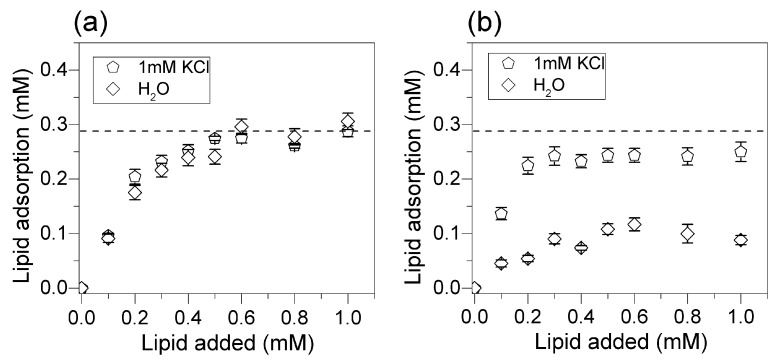
Adsorption isotherms for DODAB from films or BF onto silica. (**a**) Mean DODAB adsorption onto silica (2 mg/mL) from DODAB films. (**b**) Mean DODAB adsorption onto silica (2 mg/mL) from DODAB BF. The dashed line at 0.288 mM DODAB represents the theoretical concentration corresponding to bilayer adsorption. Each mean adsorption value and respective mean standard deviation was obtained from at least two different determinations for each element.

The mean DODAB adsorption calculated from all of the measurements derived from C, H, and N analysis yielded the adsorption isotherms on Figure 7. Figure 7a shows the mean DODAB adsorption isotherms from DODAB films in pure water or in 1 mM KCl aqueous solution, suggesting that in both cases bilayer deposition took place. This contrasted with DODAB adsorption from BF in Figure 7b showing that bilayer coating did not occur neither in pure water nor in 1 mM KCl water solution.

## 4. Discussion

Over the last three decades, our group has been attempting to quantify the adsorption of a variety of lipids onto major classes of nanostructures and surfaces such as those of polymeric or mineral nanoparticles. This task was successfully fulfilled for phospholipids since analytical methods based on spectrophotometric detection of phosphorus and phosphate allowed the reliable and sensitive determination of nanomolar concentrations of phospholipids [19,23,24,25,35]. On the other hand, the analytical determination of cationic lipids adsorbed onto NPs clearly presented the drawbacks of low sensitivity yielding huge mean standard deviations [25,26,35]. In this work, we finally determine quantitatively and with high sensitivity the in situ adsorbed amount of cationic lipids onto silica particles (see Figure 5, Figure 6 and Figure 7). The attractive electrostatic interaction between cationic lipid and deprotonated silanols drove the adsorption of the cationic lipids on silica and the deposition of the cationic bilayers [26]. However, the indirect analysis of cationic lipid in the supernatants from DODAB solubilization in micelles of a neutral surfactant incorporating the Orange G optical probe was problematic mainly over a range of small DODAB concentrations [36]. Here, the adequacy of elemental analysis to determine lipid adsorption onto silica particles in situ was demonstrated. Elemental analysis yielded systematically smaller error bars when compared to other methods available from the literature [26,36]. Below 0.5 mM of added lipid, the elemental analysis was very reliable. The interaction between DODAB and silicon wafers showed that DODAB adsorption was determined by the charge density on the charged surface, thus depending on nature and concentration of counterions in the intervening medium [34]. The cationic lipid has to displace the counterions associated to the charged surface in order to replace them. The effect of monovalent salt nature and concentration over a range of low ionic strengths (0–10 mM LiCl, NaCl, KCl, or CsCl) and at two different pH values (6.3 and 10.0) on adsorption of DODAB BF onto flat SiO_2_ surfaces was systematically evaluated by means of in situ ellipsometry [37]. High-affinity adsorption isotherms fitted by the Langmuir model indicated that adsorption maxima were consistent with bilayer deposition only around 10 mM monovalent salt at both pH values [34]. In pure water, the mean thickness of the DODAB adsorbed layer was close to zero with bilayer deposition taking place only around 10 mM ionic strength [34]. For the silica NPs, DODAB adsorption in water was also well below the amount required for bilayer deposition in contrast to the larger adsorption figures at 1 mM KCl (Figure 6 and Figure 7). Furthermore, BF were used to incorporate hydrophobic drugs, such as amphotericin B [38], or to coat hydrophobic polymeric particles [4], or to incorporate hydrophobic antimicrobial peptides, such as gramicidin D [39,40] due to their hydrophobic moieties, and eventually, hydrophobic defects. Molecular dynamic simulations for the spontaneous assembly of DODAB molecules in pure water showed that interdigitated bilayers were the preferential assemblies [33], once again suggesting the exposure of hydrophobic moieties of the interdigitated bilayer to the water medium. These hydrophobic defects might have hampered the bilayer coating on the silica particles since the silica surface is hydrophilic. Thus, in pure water, bilayer coverage was only achieved from the lipid films (Figure 6a,c and Figure 7a).

In 1 mM KCl, the results showed that DODAB adsorption from BF improved significantly, though not sufficiently to achieve a bilayer coating on all particles (Figure 7b). Possibly, counterion adsorption relaxed the high electrostatic repulsion between adjacent DODAB molecules reducing the occurrence of interdigitation and hydrophobic defects, thereby improving DODAB adsorption on silica. Other evidence for some remaining interdigitation in DODAB BF even in 1 mM KCl was the lower zeta potential values for silica/DODAB from BF dispersions as compared to those for silica/DODAB from films (Table 1). The lower zeta potencial values could also be explained by the lower amount of lipids adsorbed onto the nanoparticle surface, as demonstrated by elemental analysis. Whereas, the former were 36–38 mV, the latter were 43–55 mV, in accordance with the lower expected zeta potencial of interdigitated bilayers. In summary, in order to optimize the adsorption of cationic bilayers on hydrophilic surfaces such as those from silica particles the occurrence of hydrophobic defects and interdigitation in the bilayer should be minimized.

The experiments with DOTAP confirmed the importance of adding a small concentration of monovalent salt, such as 1 mM KCl, to achieve bilayer deposition onto all silica NPs (Figure 5c). This suggested again that cationic DOTAP bilayers in pure water might also bear hydrophobic defects or interdigitated regions to a certain extent.

## 5. Conclusions

This work showed the optimization of cationic bilayer deposition onto silica from lipid films, a procedure that advantageously skipped the preliminary step of dispersing the lipids as bilayers before promoting their interaction with silica. In addition, the determination of lipid adsorption in situ from elemental analysis considerably improved the precision of analysis and the assessment of bilayer deposition in comparison with other methods available from the literature [36]. Bilayer deposition was optimized in two ways: (1) by interacting silica with lipid films instead of pre-formed bilayer fragments, and (2) by employing 1 mM KCl instead of pure water as the intervening medium for the interaction between silica and cationic lipid. Both the low ionic strength and the use of lipid films instead of pre-formed bilayers reduced interdigitation and hydrophobic defects in the lipid assemblies [21,33,34], improving the interaction between lipid and the hydrophilic silica surface.

## Figures and Tables

**Table 1:** Sizes, zeta potentials and polydispersities for equivalent but different dispersions (numbered from 1 to 4).

Dispersion *	*Dz* (nm)	*ζ* (mV)	*P*
Silica 1	270 ± 4	−24 ± 1	0.179 ± 0.012
Silica 2	261 ± 3	−20 ± 1	0.094 ± 0.020
Silica 3	249 ± 2	−21 ± 1	0.100 ± 0.024
Silica 4	248 ± 2	−25 ± 1	0.104 ± 0.027
Silica/DOTAP film 1	284 ± 2	+45 ± 1	0.123 ± 0.028
Silica/DOTAP film 2	267 ± 3	+55 ± 12	0.096 ± 0.022
Silica/DOTAP film 3	287 ± 2	+47 ± 1	0.050 ± 0.020
Silica/DOTAP film 4	261 ± 3	+47 ± 1	0.092 ± 0.024
Silica/DODAB film 1	355 ± 19	+55 ± 3	0.267 ± 0.013
Silica/DODAB film 2	375 ± 4	+56 ± 2	0.220 ± 0.005
Silica/DODAB film 3	318 ± 3	+43 ± 3	0.136 ± 0.020
Silica/DODAB film 4	309 ± 4	+44 ± 2	0.172 ± 0.017
Silica/DODAB BF 1	251 ± 2	+36 ± 1	0.128 ± 0.021
Silica/DODAB BF 2	263 ± 3	+38 ± 1	0.204 ± 0.019

* The original dispersions at 0.5 mM lipid, 2 mg/mL silica and 24 h of interaction between lipid and silica had their supernatants diluted by 1:20 before performing the measurements. *Dz*: Z-average diameter; *P*: Polydispersity; *ζ*: Zeta potential.
